# In sepsis, 88% of bacteraemia patients are discriminated by unsupervised hierarchical cluster analysis of 5 inflammatory mediators

**DOI:** 10.1186/2197-425X-3-S1-A881

**Published:** 2015-10-01

**Authors:** KA Mosevoll, H Reikvam, HR Fanebust, H Flaaten, S Skrede, Ø Bruserud

**Affiliations:** Department of Clinical Science, University of Bergen, Bergen, Norway; Medical Department, Haukeland University Hospital, Bergen, Norway; Department of Heart Disease, Haukeland University Hospital, Bergen, Norway; Department of Clinical Medicine, University of Bergen, Bergen, Norway

## Introduction

A range of cytokines are altered during sepsis. Multiplex analysis of inflammatory mediators makes it easier to study a larger range of mediators, and combined with newer bioinformatical tools (unsupervised hierarchical clustering), this has the potential to bring new knowledge of the inflammatory process during sepsis.

## Objectives

To investigate if there are differences in expression of multiple inflammatory mediators between septic patients with and without bacteraemia during the initial phase of hospitalization, in order to identify bacteraemic patients.

## Methods

All together 80 adult, immunocompetent patients with sepsis and confirmed bacterial infection were included prospectively at Haukeland University Hospital during the first 24 hours of hospitalization at the HDU, ICU or medical department. Luminex analysis of plasma was used to analyse 35 mediators; 16 cytokines, 6 growth factors, 4 adhesion molecules and 9 matrix metalloproteinases/ tissue inhibitors of metalloproteinases. Mann-Whitney U-test were used analysing differences between the two groups were we examined both p-values with (< 0.0014) and without (p < 0.05) Bonferronis correction. We performed unsupervised hierarchical clustering of the five statistically most significant mediators using J-express software.

## Results

In 41 patients (51%) a positive blood culture was found, while the remaining 39 (49%) had proven bacterial cause by confirmed by other tests. Among these, 37 were gramnegative, 39 were grampositive, while 4 patients had mixed infections.

We found that 16 of 35 mediators showed statistical differences between bacteraemic and non-bacteraemic patients; IL1ra (p = 0.0015), IL-10 (p = 0.0009), CCL2 (p = 0.0376), CCL4 (p < 0.0001), CCL5 (p = 0.0376), CXCL8 (p = 0.0026), CXCL11 (p = 0.0468), TNFα (p < 0.0001), HGF (p = 0.0018), E-selectin (p = 0.0004), ICAM-1 (p = 0.0009), VCAM-1 (p < 0.0001), MMP-8 (p = 0.0311), TIMP-1 (p < 0.0001), TIMP-2 (p = 0.0054) and TIMP-4 (p = 0.0036).

Using unsupervised hierarchical clustering (se figure), we found that the five mediators TNFα, CCL4, E-selectin, VCAM-1 and TIMP-1 gave a reasonable good discrimination of bacteraemia patients; the patients with bacteraemia were mostly clustered in two separate groups (upper and lower cluster, 36/41 patients, 88%) showing higher levels of the mediators. Only 5 (12%) of the bacteraemic patients were clustered in the middle cluster, while most of the non-bacteraemia patients (28/39, 72%) were clustered in this group (Chi-square p < 0.0001).

## Conclusions

We find higher levels of inflammatory markers in sepsis patients with bacteraemia, and hierarchical clustering may aid early identification of patients with bacteraemia.

Upper and lower cluster contains most bacteraemia patients (red) showing higher levels of inflammatory mediators (green). Middle cluster with lower levels of mediators (blue) contains most patients without bacteraemia. Mediators are clustered horizontally and patients vertically.

**Figure 1:**
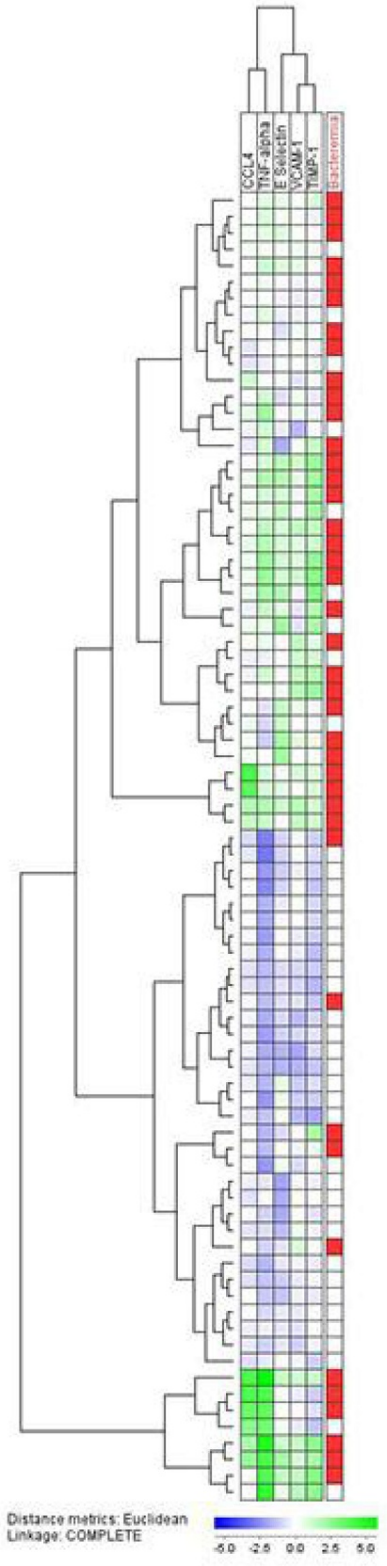
**Unsupervised hierarchical cluster analysis**.

